# Supplementary material for: Stomatal patterning is differently regulated in adaxial and abaxial epidermis in Arabidopsis
Source: J Exp Bot. 2024 Aug 19;75(20):6476–88. doi: 10.1093/jxb/erae354 (PMC11523041; doi:10.1093/jxb/erae354)
Supplement: erae354_suppl_Supplementary_Figures_S1-S3_Table_S1 [file erae354_suppl_supplementary_figures_s1-s3_table_s1.pdf]

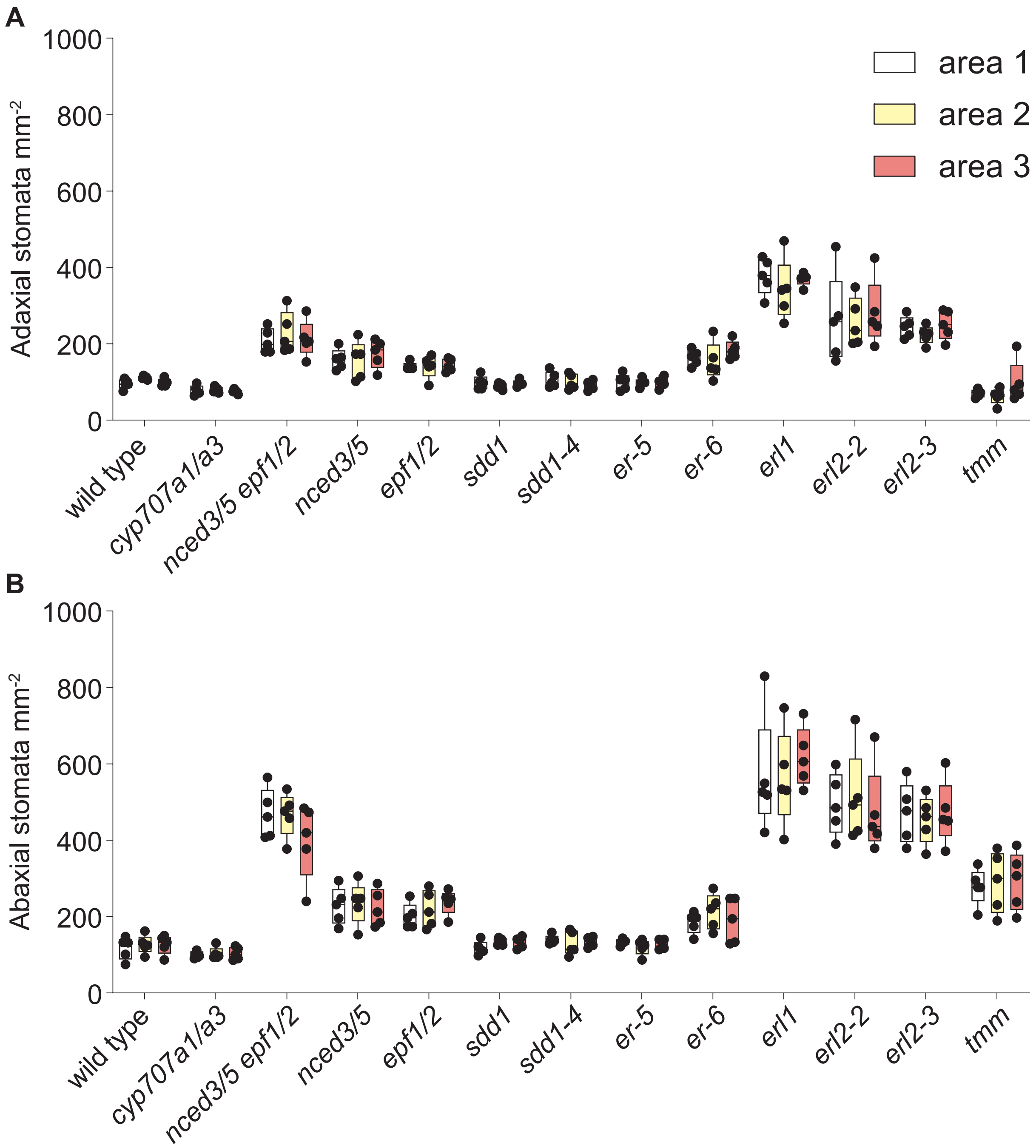

Supplementary Figure S1. Comparison of stomatal density in three different areas sampled from the same imprint in the adaxial (A) and abaxial (B) leaf side. The boxes represent the 25th and 75th percentiles, with the median indicated with the horizontal line; the whiskers show the range of values. Solid dots represent individual plants, n = 5. Two-way ANOVA with genotype and area as factors showed no significant interaction or effect of sampled area on stomatal density, only the effect of genotype was significant.

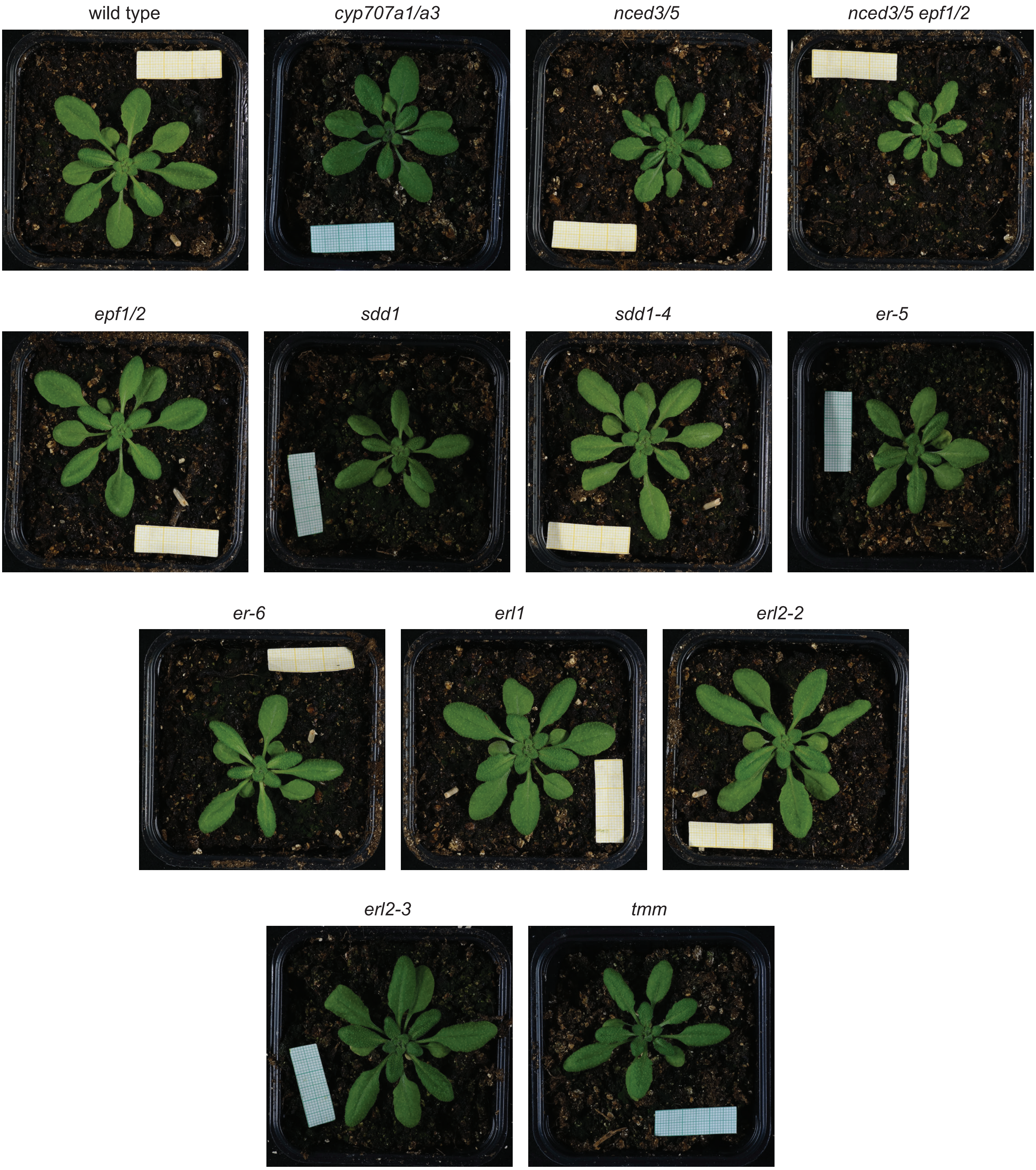

Supplementary Figure S2. Representative images of 4-week-old plants.

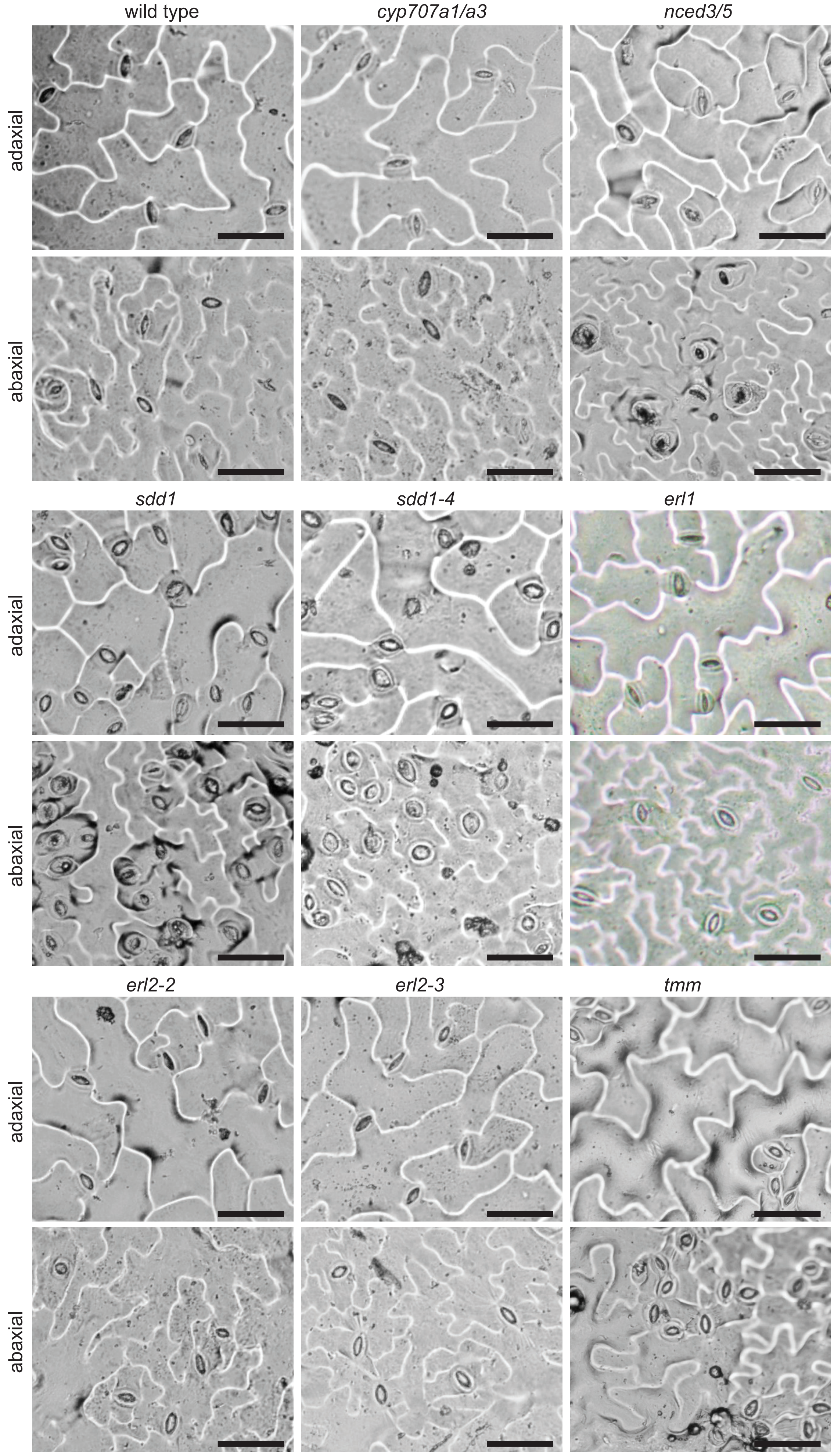

Supplementary Figure S3. Representative images of abaxial and adaxial stoma-tal impressions. The scale bar represents 50 µm.

Supplementary Table S1. Primers used for genotyping studied mutants

Allele name	Line name	Border primer	Left primer (LP)	Right primer (RP)	Genotyping principle
nced3-2	GK-129B08	ATATTGACCATCA TACTCATTGC	GTCAGCCACGAGA AGCTACAC	TTCACCGGTTTTGAGA TTCAG	Product with LP + RP from wild-type allele, and with border primer + RP from mutant allele
nced5-2	GK-328D05	ATATTGACCATCA TACTCATTGC	TAACACCAAACCCA ACCAAAC	TGACTCAACCCAAACC ATCTC	Product with LP + RP from wild-type allele, and with border primer + RP from mutant allele
epf1-1	SALK_137549	ATTTTGCCGATTT CGGAAC	GGTGCATGTTCGA CACTCTTC	CATGGTCATGTCCCG GAGAAGC	Product with LP + RP from wild-type allele, and with border primer + RP from mutant allele
epf2-2	GK-673E01	ATATTGACCATCA TACTCATTGC	ATGACGAAGTTTGT ACGCAAG	AATCTGATTCGGTCGT GTC	Product with LP + RP from wild-type allele, and with border primer + RP from mutant allele
cyp707a1-1	SALK_069127	ATTTTGCCGATTT CGGAAC	CATGAACGTATTGG GTTTTGG	TCCTGATATTGAATCC ATCGC	Product with LP + RP from wild-type allele, and with border primer + RP from mutant allele
cyp707a3	SALK_101566	ATTTTGCCGATTT CGGAAC	GTTCCTGGAAGATT AATCGGC	ACGTGCTCTCGTCACT CTCTC	Product with LP + RP from wild-type allele, and with border primer + RP from mutant allele
pyr1-1	Q169stop		TCGGTTCGAGAAA GAGAATCG	CGTCATTCTCATCATA AGAAAATGG	PCR product digested with HpyCH4V (New England Biolabs), cuts wild-type but not mutant product
pyl1-1	SALK_054640	ATTTTGCCGATTT CGGAAC	TGCCAATTTTCAGA CATTAAGC	AACCATGCCTTCCGAT TTAAC	Product with LP + RP from wild-type allele, and with border primer + RP from mutant allele
pyl2-1	GT_2864	CCGTTTACCGTTT TGTATATCCCG	ATGAGCTCATCCCC GGCCG	TTCATCATCATGCATA GGTGCAG	Product with LP + RP from wild-type allele, and with border primer + RP from mutant allele
pyl4-1	SAIL_517_C08	GCCTTTTCAGAA ATGGATAAATAG CCTTGCTTCC	TTCCAATCGTTCCA AATATCG	TAAGACTCGACAACGA CGGTC	Product with LP + RP from wild-type allele, and with border primer + RP from mutant allele

pyl5	SM3_3493	TACGAATAAGAGCGTCCATTTTAGAGTGA	AAACACAAAGCCTTCACATCC	AAGTTTTGTGAATCCCCCAAC	Product with LP + RP from wild-type allele, and with border primer + RP from mutant allele
pyl8-1	SAIL_1269_A02	GCCTTTTCAGAAATGGATAAATAGCCTTGCTTCC	AGAGAGTGGAACCCCATGATC	TTCTTCTTCTTCCTTCATGCG	Product with LP + RP from wild-type allele, and with border primer + RP from mutant allele
er-5	GK-182D08	ATATTGACCATCATACTCATTGC	AGCAAAAGATGCACAAAGAGG	CCTGATCATCTGAGCTCTTGC	Product with LP + RP from wild-type allele, and with border primer + RP from mutant allele
er-6	GK-364C05	ATATTGACCATCATACTCATTGC	AATATCAAAGGTCCAATCCCG	ATGCACAATACCAAAACCTGC	Product with LP + RP from wild-type allele, and with border primer + RP from mutant allele
erl1	GK-109G04	ATATTGACCATCATACTCATTGC	TTTCCAATCATGATGTTGCAG	CAAACAATTGCTCCAGCTTTC	Product with LP + RP from wild-type allele, and with border primer + RP from mutant allele
erl2-2	SALK_015275C	ATTTTGCCGATTTCGGAAC	AATGACACATCGCTGAGAAGG	TATCTCCATGGCAACAAGCTC	Product with LP + RP from wild-type allele, and with border primer + RP from mutant allele
erl2-3	GK-486E03	ATATTGACCATCATACTCATTGC	TATCTCCATGGCAACAAGCTC	AATGACACATCGCTGAGAAGG	Product with LP + RP from wild-type allele, and with border primer + RP from mutant allele
sdd1	GK-627D04	ATATTGACCATCATACTCATTGC	TCTTTTGTTGCTGAAAAAGGC	ACACGGTGTCCTCTGATGAAG	Product with LP + RP from wild-type allele, and with border primer + RP from mutant allele
sdd1-4	GK-693D08	ATATTGACCATCATACTCATTGC	GTTGAATCTCTTGCGGAAATG	TCTTTTGTTGCTGAAAAAGGC	Product with LP + RP from wild-type allele, and with border primer + RP from mutant allele
tmm	SALK_115723C	ATTTTGCCGATTTCGGAAC	ATCTAGGGCCCAACACAAGAC	AATTGGTTGAGCCGGTTAATC	Product with LP + RP from wild-type allele, and with border primer + RP from mutant allele

stkr1	SALK_115723C	ATTTTGCCGATTTCGGAAC	TTGAGAAAAGTATGGCCAAGTG	AAGCTTGGCGGAAAATCTAAG	Product with LP + RP from wild-type allele, and with border primer + RP from mutant allele
